# Comparing the functional impact of knee replacements in two cohorts

**DOI:** 10.1186/1471-2474-15-145

**Published:** 2014-05-05

**Authors:** Jingbo Niu, Michael Nevitt, Charles McCulloch, James Torner, C Elizabeth Lewis, Jeffrey N Katz, David T Felson

**Affiliations:** 1 From the Clinical Epidemiology Unit, Boston University School of Medicine, 650 Albany Street, Suite X200, Boston, MA 02118, USA; 2 Department of Epidemiology and Biostatistics, University of California, San Francisco, USA; 3 Department of Epidemiology, University of Iowa, Iowa City, Iowa, USA; 4 Department of Preventive Medicine, University of Alabama, Birmingham, England; 5 Department of Orthopedic Surgery and the Division of Rheumatology, Immunology and Allergy at the Brigham and Women’s Hospital, Boston, USA

**Keywords:** Total knee replacement, Function, Epidemiology

## Abstract

**Background:**

To examine if different rates of total knee replacement (TKR) in two similar cohorts with symptomatic knee osteoarthritis (OA) were associated with different functional impact of disease.

**Methods:**

Subjects from the Multicenter Osteoarthritis Study (MOST) and the Osteoarthritis Initiative (OAI), persons with or at high risk of OA, had knee radiographs, completed Western Ontario and McMaster Universities Osteoarthritis Index (WOMAC) surveys and had TKRs confirmed at each visit. At each visit, subjects were defined as having symptomatic OA (SxOA) if ≥ one knee had pain and radiographic OA or if they had a TKR. WOMAC function scores at each visit were compared by analysis of covariance adjusting for age, sex, body mass index, race, site, depression, comorbidity, painful leg joints and knees affected. Post-TKR function scores were imputed to estimate scores that would have been present without TKR.

**Results:**

Subjects with SxOA (n > 750 in MOST and in OAI) had a mean age 66 to 67 years; most were women and were White. Subjects were followed 4–5 years. Among those with SxOA, more TKRs were done in MOST (35%) than OAI (19%). Adjusted mean WOMAC function (0–68, 68 = worst) improved from 26.9 to 21.9 in MOST and from 24.5 to 22.0 in OAI (difference between MOST and OAI in change in WOMAC function, p = .01). Estimates of function without TKRs showed function would not have changed in MOST (23.2 at baseline to 22.4).

**Conclusions:**

Functional status of subjects with knee OA in MOST improved more than in OAI, probably because of higher rates of TKRs. The decline suggests that TKR diminishes the functional impact of OA in the community.

## Background

Knee osteoarthritis (OA) is a painful and disabling disorder affecting approximately 6% of US adults [1]. Medical and rehabilitative treatments are limited and increasingly, persons affected by disease have sought total knee replacement (TKR). Of persons undergoing TKR 70-90% have a marked reduction in pain and improvement in reported function within 12 months after the surgery [2], but a substantial minority do not experience improvement [3,4]. Rates of TKR have increased dramatically in the last 25 years, with the numbers rising over 8-fold since 1980 [5] in the U.S. There has also been a marked rise in the rates of TKR in the U.K. This increase has continued to occur in the 21^st^ century with rates rising even in the last 5 years [6-9].

In two cohorts constituted of persons with or at high risk of symptomatic knee OA, we discovered that over time, participants in one of the cohorts underwent many more TKR’s than those in the other cohort. This difference created a natural experiment to determine whether among large cohorts containing numerous persons with symptomatic knee osteoarthritis, a higher rate of TKR in one cohort would lead to a reduction in the functional impact of osteoarthritis compared with the other group.

While numerous studies have shown that individuals undergoing TKR on average experience an improvement in function [2] even compared with those who have OA but do not undergo TKR [10], we are unaware of any studies which have addressed whether the number of TKR’s in a group with OA are sufficient to affect the overall impact of disease on function limitation in all those with disease. Even if those with TKR have better function than those with knee OA without TKR, the difference is not necessarily a large one and may not be sufficient to have an effect on the population of persons with knee OA until a large percentage of persons with knee OA get replacements. In two cohorts with very different rates of TKR, we tested the overall functional impact of TKR on function.

## Methods

### Patients and methods

We focused on data from two community based cohort studies, the Multicenter Osteoarthritis Study (MOST) and the Osteoarthritis Initiative (OAI). Both studies enrolled persons either with or at high risk of symptomatic knee OA with the goal of identifying risk factors for incident and progressive knee osteoarthritis. Both studies followed subjects for several years. In both cohorts, a large proportion of persons with knee OA obtained knee replacements during the follow-up. Informed consent was obtained from all participants.

### MOST

MOST recruited persons with or at high risk of knee osteoarthritis from the communities of Birmingham, Alabama and Iowa City, Iowa. Subjects with bilateral TKR at baseline were excluded. 3,026 subjects aged 50–79 at baseline were studied at baseline, 30 and 60 months. At each visit, subjects filled out the Western Ontario and McMaster Universities Osteoarthritis Index (WOMAC) survey for knees, were asked about pain in other joints, and completed questionnaires on depressive symptoms (CES-D) and co-morbidities (Charlson co-morbidity score). Weight and height were measured and PA and lateral weight bearing radiographs obtained. Radiographs were read by a pair of experienced readers, with disagreement resolved by adjudication [11]. Self-reported TKR was confirmed by radiographs and/or medical records.

For the purposes of this investigation, persons with symptomatic knee osteoarthritis were identified separately at each visit. A person was defined as having this entity if they had one knee with knee pain on most days reported at the clinic visit and radiographs showing osteoarthritis (Kellgren & Lawrence grade 2 or greater) or if the person had a TKR at the time of the visit. To be consistent in defining symptomatic OA at all visits, we applied this definition to the baseline examination also. The symptomatic knee osteoarthritis status of a subject might change in either direction from one visit to the next. For example, a person could develop new symptomatic knee osteoarthritis if their knee pain was newly reported or their symptomatic knee OA could go away if their knee pain present at the earlier visit, resolved by the later visit (knee pain is often episodic). The protocol for the MOST Study was approved by IRBs at Boston University, the University of Iowa, the University of Alabama, Birmingham and the University of California, San Francisco.

### Osteoarthritis Initiative (OAI)

In the OAI Study subjects with or at high risk of knee osteoarthritis were recruited from four communities: Columbus, Ohio; Providence, Rhode Island; Baltimore, Maryland and Pittsburgh, Pennsylvania. Eligibility for OAI was similar to that of MOST with a few exceptions: in OAI, the risk factors permitting eligibility to the study were broader and age extended to those as young as age 45. Assessments were similar to MOST except that they were done yearly. Other differences included:

1. Lateral weight bearing radiographs were not acquired in OAI.

2. Subjects reported WOMAC function for each knee.

In OAI, knee radiographs were read and adjudicated by the same team as in MOST using the same protocol. We also used the same definition for symptomatic knee osteoarthritis. Racial/ethnic background was determined by subject self-report.

The higher (worse) WOMAC function score of the two knees was used as the WOMAC function of a subject [12].

Of those with knee surgeries, 13 in the combined OAI and MOST studies had partial knee replacements and for analysis purposes, we lumped these with TKRs. The OAI protocol was approved by the IRBs of all participating institutions including the Universities of Maryland, Pittsburgh, and California, San Francisco, the Ohio State University and that of Memorial Hospital, Rhode Island.

### Defining the impact of knee osteoarthritis

To assess functional status related to knee OA, we used the WOMAC function scale in which subjects are asked how much difficulty they have doing each of 17 activities because of their knee [13]. Higher WOMAC function scores (0–68 scale) signify worse function. We also examined WOMAC pain scores (0–20 scale, higher scores signify worse pain).

### Statistical analyses

To create comparable subjects at each time point in each study, we limited analyses of both cohorts to those aged 55–79 years at each visit. We did this because knee replacements are rare in persons under age 55. A person under age 55 at baseline would enter into our group for analysis purposes at the exam at which they reached age 55 and others would leave the cohort when their age became greater than 79 years. For MOST, we looked at three groups of subjects with symptomatic osteoarthritis: one at baseline, another at 30 months and another at 60 months. For OAI, we used the same approach, only applied it at each biannual visit through the 48 month visit. At each time point, we examined which subjects met criteria for symptomatic osteoarthritis and were in the appropriate age window.

If removal of those with severe symptomatic knee OA (because they got TKR’s) were to account for improvement in functional status, we would anticipate that those undergoing TKR would have substantially worse function and pain scores prior to surgery than those not undergoing this surgery. To examine this question, we focused on MOST or OAI examinations just prior to subjects’ first TKR. We compared their WOMAC function and pain scores from those pre-KR visits to others in these cohorts who had symptomatic knee osteoarthritis.

We estimated the crude mean WOMAC function score at each time point, then used an ANCOVA model to test whether there was a significant difference in adjusted WOMAC scores over time after adjusting for factors known to affect function in those with OA or in those after TKR [14-16], age, sex, BMI, race, clinical site, CES-D score, co-morbidity, number of painful joints in lower limbs other than knees, and number of knees with symptomatic OA. GEE was used to adjust for correlation between repeated measures within a subject.

To examine whether differences in functional status of SxOA emerged over time in MOST vs. OAI, we carried out the same ANCOVA analyses adding study (MOST or OAI) as an indicator variable and in addition added a Time X study interaction term which tested whether the two cohorts differed in the extent of change in WOMAC function score over the course of follow-up.

To estimate the functional status that would have been present in each cohort if there were no TKR’s during follow-up, we examined subjects with symptomatic osteoarthritis at baseline and not lost to follow-up. For those who obtained TKR’s during the follow-up, we imputed WOMAC function scores at these post TKR visits based on the covariates age, sex and race as well as their baseline BMI, knee pain score, function score and Kellgren & Lawrence grade. We used Markov Chain Monte Carlo methods (MCMC) method to created five multiple imputed datasets. The mean WOMAC function scores were pooled across the imputed datasets along with adjusted variance and taking the uncertainty introduced by the imputation into account. We then described the distribution of WOMAC function scores at each time point, first using actually observed scores and again using the imputed scores of those with a TKR.

We carried out a secondary analysis in which instead of self-reported function as a measure of disease impact, we examined WOMAC pain score.

## Results

**Figure 1:**
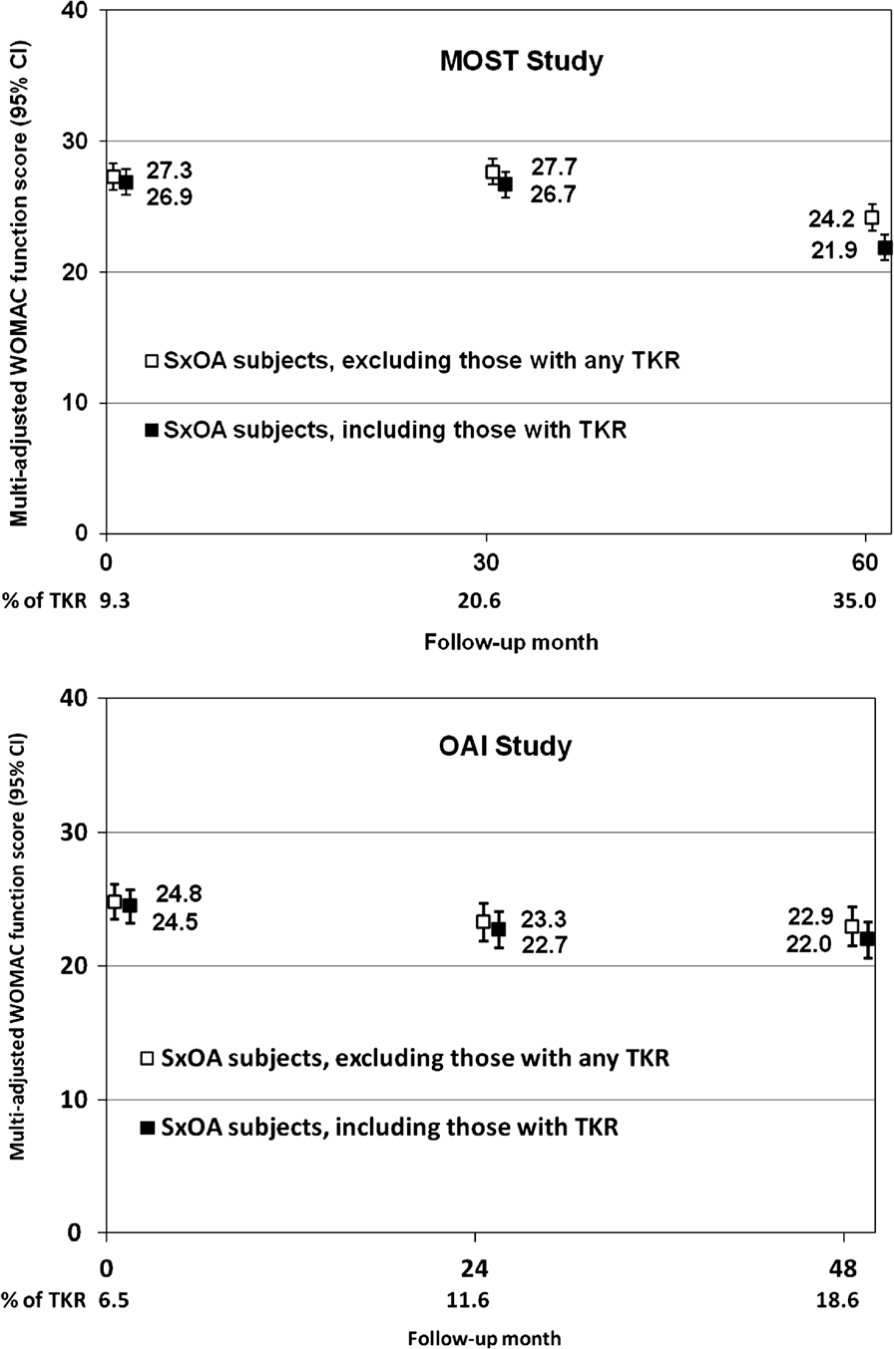
Secular trend of mean WOMAC function score among subjects with symptomatic knee OA#.

In each of the MOST and OAI cohorts there were over 750 persons with symptomatic knee osteoarthritis at each examination. Groups were similar both across time points and across cohorts (see Table 1) with respect to being in their late 60’s on average, being mostly women and having a mean BMI in the obese range. Many subjects had painful joints outside of their knees. More subjects in MOST got their knees replaced than did OAI subjects. The cumulative frequency of at least one TKR in MOST subjects with symptomatic knee OA was 35.0% over 5 years (vs. 9.3% frequency in the same group at study baseline). In OAI, the cumulative frequency was only 18.6% over 4 years (see Figure 1) and, of this group, 6.5% had TKR at baseline. Also the WOMAC function among those with symptomatic knee osteoarthritis was worse among subjects with symptomatic osteoarthritis in MOST than in OAI (for example, at baseline the crude WOMAC function score was 24.3 in MOST vs. 19.6 in OAI).

**Table 1:** Characteristics of participants with symptomatic knee OA in each cohort at each time point

	**MOST study**			**OAI study**		
	**Baseline (N = 824)**	**30 month (N = 818)**	**60 month (N = 765)**	**Baseline (N = 880)**	**24 month (N = 793)**	**48 month (N = 762)**
Age, mean (SD), year	66.1 (6.4)	66.6 (6.7)	67.3 (6.8)	66.1 (6.7)	66.3 (6.9)	66.9 (7.1)
N (%) of women	541 (65.7)	527 (64.4)	512 (66.9)	515 (58.5)	486 (61.3)	451 (59.2)
N (%) of race						
Whites	679 (82.4)	672 (82.2)	631 (82.5)	633 (71.9)	627 (79.1)	604 (79.3)
Blacks	137 (16.6)	139 (17.0)	118 (15.4)	219 (24.9)	147 (18.5)	135 (17.7)
Others	8 (1.0)	7 (0.9)	16 (2.1)	28 (3.2)	19 (2.4)	23 (3.0)
BMI, mean (SD), kg/m^2^	32.6 (6.6)	32.5 (6.5)	32.9 (6.8)	29.9 (4.7)	30.1 (4.8)	30.4 (4.9)
CES-D, mean (SD)	8.7 (8.5)	8.0 (7.6)	7.8 (7.6)	7.1 (6.9)	6.9 (7.3)	7.5 (7.5)
Charlson comorbidity score, mean (SD)	0.7 (1.0)	0.8 (1.2)	0.9 (1.4)	0.5 (0. 9)	0.6 (1.0)	0.5 (1.0)
# of painful joints in lower limb other than knee, mean (SD)	2.0 (1.9)	1.4 (1.7)	1.9 (1.8)	1.0 (1.4)	0.9 (1.3)	0.9 (1.3)
N (%) with KR						
Unilateral KR	77 (9.3)	139 (17.0)	167 (21.8)	57 (6.5)	88 (11.1)	123 (16.1)
Bilateral KR	0 (0)	29 (3.6)	101 (13.2)	0 (0)	4 (0.5)	19 (2.5)
N (%) with partial and total KR						
Partial KR	3 (0.4)	9 (1.1)	10 (1.3)	0 (0.00)	0 (0.00)	2 (0.3)
Total KR	74 (9.0)	158 (19.3)	257 (23.6)	57 (6.5)	92 (11.6)	139 (18.2)
Partial KR and total KR	0 (0)	1 (0.1)	1 (0.1)	0 (0)	0 (0)	0 (0)
WOMAC function, mean (SD)	24.3 (11.7)	23.0 (12.5)	19.0 (12.2)	19.6 (12.6)	17.5 (12.4)	17.0 (11.9)
Maximal WOMAC knee pain scale, mean (SD)	7.3 (3.7)	6.8 (3.9)	5.6 (3. 9)	6 (3.9)	5.6 (3.8)	5.5 (3.7)

When we examined the pre-KR status of persons who later underwent TKR’s, we found that their WOMAC function and pain scores were worse than those with symptomatic OA who did not undergo TKR’s (for function in MOST, crude score 28.4 vs. 22.9, p < .0001; in OAI, 24.8 vs. 18.0, p < .0001; for pain in MOST, crude score 8.6 vs. 6.9, p < .0001 and for pain in OAI, crude score 7.8 vs. 5.6, p < .0001).

In MOST we found an improvement in the adjusted WOMAC function score (p < .0001) over time in persons with symptomatic osteoarthritis, a group which as noted above includes persons with TKR (see Figure 1top). To determine whether this was partially accounted for by removal of those with the most severe disease leaving only those with milder disease, we excluded those subjects with TKR (see Figure 1top). We found a drop in the WOMAC function score, suggesting that at least at 60 months, those with the most severe knee osteoarthritis had TKRs.

The improvement in physical functional status among those with symptomatic knee OA was not as marked in the OAI cohort (see Figure 1bottom). Even so, the WOMAC function score improved significantly (p < .0001) and there was a decline in WOMAC score even when we excluded those who had TKR.

When we analyzed the combined group, we found that those in MOST had an adjusted decrease (improvement) in WOMAC function score of 1.0 per year (95% CI −1.2, −0.8) and it was 0.6 per year for OAI (95% CI −0.9, −0.4). These changes were unadjusted for WOMAC function differences between MOST and OAI. We then tested to see if the WOMAC function scores of MOST and OAI were different and if the changes over time in function between the studies was different (see Table 2) after adjusting for differences in function scores between MOST and OAI. MOST subjects with SxOA had a higher WOMAC function score than OAI subjects (p < .001) but after adjusting for this and other factors, WOMAC function scores improved more in MOST than in OAI (p = .01 for interaction of time X study). When we removed persons with TKR from both cohorts, we no longer found differences in change scores in OAI vs. MOST.

**Table 2:** Secular trend of WOMAC function score in two studies with interaction between study and time

		Annual WOMAC function change (95% CI)*	**P value**
**SxOA subjects**			
Visit, year		−0.64 (−0.88, −0.39)	<.0001
Study	MOST	3.15 (2.14, 4.16)	<.0001
Study	OAI	1.0 (referent)	
Visit* study	MOST	−0.39 (−0.70, −0.08)	0.01
Visit* study	OAI	1.0 (referent)	
**SxOA subjects, excluding those with TKR**			
Visit, year		−0.50 (−0.726 -0.23)	0.0008
Study	MOST	3.24 (2.20, 4.29)	<.0001
Study	OAI	1.0 (referent)	
Visit* study	MOST	−0.08 (−0.41, 0.25)	0.64
Visit* study	OAI	1.0 (referent)	

*Adjusted for age, sex, BMI, race, clinical site, CES-D, co-morbidity, number of painful joints in lower limbs other than knees, and number of knees with symptomatic OA.

**Figure 2:**
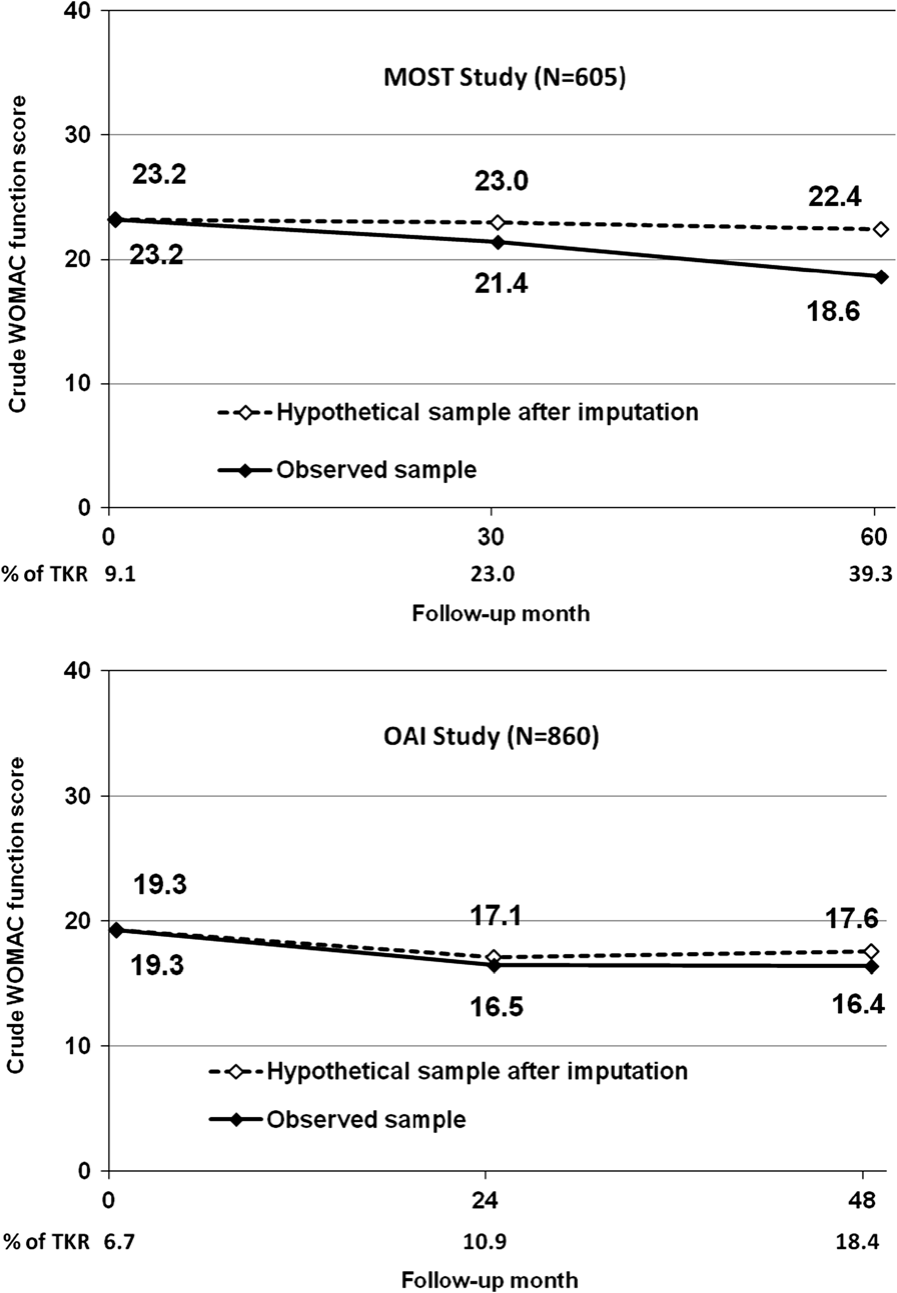
WOMAC function score observed in those with symptomatic OA and WOMAC function score imputed in those with symptomatic OA if there were no TKR#.

To evaluate the effect of TKR on the average WOMAC function score in persons with symptomatic knee osteoarthritis, we imputed WOMAC physical function scores for subjects at the time of their replacements so that we could project a WOMAC function score if they had not had replacements (see Figure 2). In MOST, the imputed WOMAC physical function scores did not change significantly over time, starting at 23.2 and ending at 22.4. In the OAI, there was an improvement in WOMAC physical function score in the imputed sample from 19.3 to 17.6.

Using WOMAC pain as an outcome, a similar trend for improvement was seen in both the MOST study (adjusted pain score fell from 8.2 to 6.6, p < .0001) and in the OAI (adjusted pain score fell from 7.4 to 7.0, p = .004). In similar analyses to those examining WOMAC function, we found differences in pain change between MOST and OAI, changes that were not present when we removed persons with TKR’s.

## Discussion

Our findings from two large cohort studies that included persons with and at risk of symptomatic osteoarthritis showed that the average physical function score for those with symptomatic osteoarthritis improved more in the cohort with the higher rate of TKRs. Our results suggest that the functional impact of osteoarthritis will decline modestly when the proportion of persons with knee osteoarthritis with knee replacements rises dramatically. Starting at baseline, the new TKR rate differed by 14% of those with disease in MOST vs. OAI (26% vs. 12%) and the WOMAC function score fell by 2.5 points more in MOST.

While the rates of TKR in the population have increased markedly including in the 2000s [6], there are no population data on the proportion of those potentially eligible for replacement who have actually had the surgery. The cohorts we studied are drawn from the community (and not from clinics) but are not necessarily representative of the populations from which they were drawn. Even so, these data provide a valuable projection of the effects of different TKR rates on the functional status of populations with knee OA.

Previous studies have shown that persons after knee replacement have an average improvement in physical function not experienced by age and gender matched controls without disease [17] and that they experience relative improvements in function compared to matched persons with symptomatic OA [10]. These studies compared those with TKR to those without it, and our study extends these findings by studying the entire population likely to be affected by increased rates of this procedure and examining the procedure’s overall impact on this population.

What else might account for our findings? First, in both the OAI and MOST, there might have been regression to the mean with subjects entering the study at a time when they were experiencing severe pain and functional limitation with a natural subsequent improvement. Regression to the mean may well account for the improvement in function at the beginning of the study in those without TKR but it probably does not explain our findings. When we imputed the function scores of those who underwent TKR we saw constancy, especially in MOST, suggesting that our results could not reasonably be explained by regression to the mean (see Figure 2). If there were regression to the mean, imputed scores would have shown improvement. Also, regression to the mean would have led to improvements in both cohorts and yet the improvement was significantly greater in MOST, the cohort with the higher rates of TKR. Another explanation for the improvement might be improved medical or rehabilitative therapy for knee osteoarthritis, but there has been no effective new treatment widely adopted by patients since the beginning of these studies. Further, we found no change over time in the proportion of subjects using analgesics or NSAIDs for OA in either cohort (data not shown).

OAI subjects with symptomatic OA had slightly milder OA at study onset probably because of study eligibility differences. Potential subjects with no knee pain or obesity and with only hand OA and with parents or siblings with knee replacement were eligible for OAI but not for MOST.

One might speculate that the high rates of knee replacement in MOST are due in part to the locations of these cohorts, situated in small cities with prominent academic medical centers where TKR may be more available than in the larger, more diverse cities where OAI was situated. Also, OAI had a modestly higher proportion of African Americans who generally have lower rates of TKR utilization. The differences in the rates of TKR in MOST and OAI cohorts may be informative in terms of the ultimate effects of this surgery. They suggest as TKR rates rise further that the improvement in physical function experienced by symptomatic knee osteoarthritis patients will be greater (see Figure 1).

There are a number of limitations to our work. First, other factors could have accounted for the differences in these cohorts. In MOST, the average function scores were worse than in OAI and regression to the mean could have been more marked there. Our analysis of change adjusted this overall difference and showed a significant difference with MOST subjects improving more than OAI subjects even after adjustment for differences in baseline function (see Table 2). Another potential limitation is loss to follow-up. In both studies over 85% of subjects initially recruited were tracked with respect to symptomatic knee osteoarthritis status and functional impact. Most of those lost in MOST were contacted by phone and the proportion with TKR was similar to those followed and who completed a WOMAC survey (37% vs. 42%). suggesting that our estimates of the proportion with TKR in the entire cohort are accurate. The follow-up times were different in the two cohorts and this could have introduced differences in WOMAC change. An additional limitation is that we do not know in either of these studies how TKR’s were allocated and whether they were carried out in those most likely to experience functional improvement. Compared to rates and impact in MOST, it is conceivable that lower rates of TKR would lead to even larger improvements in function if their allocation were optimal.

Our data suggest that TKR surgery has a broad effect on the functional impact of knee osteoarthritis in the community. This finding may be useful in assessing the advantages and disadvantages of the large societal investment in TKR. The overall effects of TKR increase on the larger population with knee OA were small---one WOMAC function point yearly was gained by the increase in TKR rates in MOST compared with OAI. The estimate of minimal clinically important impact for WOMAC physical function after TKR is 14, and the population effect seen is much smaller than this. While considerable research has examined the extent of change in functional status (e.g. as measured by the WOMAC) that is clinically important for individuals, the extent of change across populations that is meaningful and relevant to policy decisions merits investigation. Even though our findings suggest a favorable population effect of TKR, they do not argue for more TKR’s without consideration of clinical appropriateness.

## Conclusions

In summary, based on work from two large cohorts drawn from communities in the United States in which there has been a substantial difference in rates of TKR, the group with the higher rate of TKR has had a greater improvement in OA related functional status. The best explanation for this is that the high rate of TKR’s has led to a diminishing functional impact of disease.

## Abbreviations

TKR: Total knee replacement; OA: Osteoarthritis; MOST: Multicenter Osteoarthritis Study; OAI: Osteoarthritis Initiative; WOMAC: Western Ontario and McMaster Universities Osteoarthritis Index; SxOA: Symptomatic OA; MCMC: Markov Chain Monte Carlo methods.

## Competing interests

The authors declare that they have no competing interests.

## Authors’ contributions

Conception and Design: JN, DTF. Analysis and interpretation of data: JN, MN, CM, JT, CEL, JNK. Drafting Article: JN, DTF. Critical revision of article: MN, CM, JT, CEL, JNK. Final Approval: all authors.

## Pre-publication history

The pre-publication history for this paper can be accessed here:

http://www.biomedcentral.com/1471-2474/15/145/prepub
